# Association between systolic blood pressure and uric acid in Chinese children and adolescents with idiopathic short stature: a cross-sectional study

**DOI:** 10.1038/s41371-020-0362-0

**Published:** 2020-06-09

**Authors:** Shuang Kou, Mei Zhang, Baolan Ji, Qianqian Zhao, Yanying Li, Hui Pan, Bo Ban, Ping Li

**Affiliations:** 1 Department of Clinical Medicine, Jining Medical University, 16 Hehua Road, Beihu New District, Jining, 272067 Shandong China; 2 Department of Endocrinology, Affiliated Hospital of Jining Medical University, Jining Medical University, Jining, 272029 Shandong China; 3 Chinese Research Center for Behavior Medicine in Growth and Development, Jining, 272029 Shandong China; 4 Key Laboratory of Endocrinology of National Health and Family Planning Commission, Department of Endocrinology, Peking Union Medical College Hospital, Chinese Academy of Medical Science and Peking Union Medical College, Beijing, 100730 China

**Keywords:** Hypertension, Endocrine system and metabolic diseases

## Abstract

The purpose of this study was to investigate the relationship between systolic blood pressure (SBP) and uric acid (UA) in patients with idiopathic short stature (ISS). The present study was a cross-sectional study. A total of 210 Chinese children and adolescents with ISS were included, and their anthropometrics and biochemical parameters were measured. Growth hormone peak levels were assessed after provocation tests with L-dopa and insulin. The univariate analysis results showed a significant positive association between UA and SBP levels (*P* < 0.001). Furthermore, a non-linear relationship was detected between UA and SBP. In multivariate piecewise linear regression, the inflection point of UA was 4.13 mg/dl (95% CI 3.28, 6.65; *P* = 0.03), the levels of SBP increased with the increase in UA when the UA level was >4.13 mg/dl (*β* 2.63, 95% CI: 0.94, 4.31; *P* = 0.002). However, we did not observe a significant relationship between UA and SBP when the UA level was <4.13 mg/dl (*β* −2.72, 95% CI −6.89, 1.45; *P* = 0.202). Our study found a nonlinear relationship between UA and SBP in Chinese children and adolescents with ISS and showed that SBP levels were associated positively with the rise of UA levels when the UA levels reached the inflection point.

## Introduction

Short stature refers to individuals in a similar living environment and of the same race, same sex and age who are two standard deviations lower than the average height of the normal population. Despite the short stature, for many affected children and adolescents, there are no clear reasons for the identified growth problems. This heterogeneous group of children and adolescents is often described as having ISS. ISS is one of the major causes of short stature [1], and our previous studies have shown that ISS accounts for 30% of all short stature aetiologies [2] and may cause many physical and psychological adverse effects.

Short stature is considered a risk factor for adult cardiovascular diseases (CVDs), such as coronary heart disease and hypertension [3, 4]. The causes of CVD are often complex and multifactorial and may be rooted in childhood [5]. Worldwide, one of the most common causes of death and disability-adjusted life-years is hypertension [6]. Previous literature has reported that shorter adult heights were associated with higher blood pressure (BP) [7]; Korhonen et al. reported that adults with short stature had higher CVD risk and higher BP (especially SBP) than the common population [8]. Adult primary hypertension is likely derived from risk factors already present in childhood [9]. Therefore, the identification of these cardiovascular risk factors is necessary for the standard treatment of short stature in children and adolescents.

UA is the final product of purine metabolism. Dietary intake affects the production and metabolism of UA to a large extent. Picky eating, partial eating and the excessive intake of high-sugar drinks are poor eating habits that are common in short stature children and adolescents and that may lead to an increase in uric acid levels [10]. An elevated UA level is a risk factor for metabolic syndrome, which may include obesity, dyslipidaemia, hypertension, impaired glucose tolerance and other metabolic disorders [11]. Furthermore, hyperuricaemia is a recognised risk factor for hypertension in adults and children [12, 13].

Therefore, it is necessary to evaluate the relationship between UA and SBP in ISS children and adolescents. However, to the best of our knowledge, few studies have focused on this issue. The aim of this study was to examine the relationship between SBP and UA in Chinese children and adolescents with ISS.

## Methods

### Study population

**Fig. 1:**
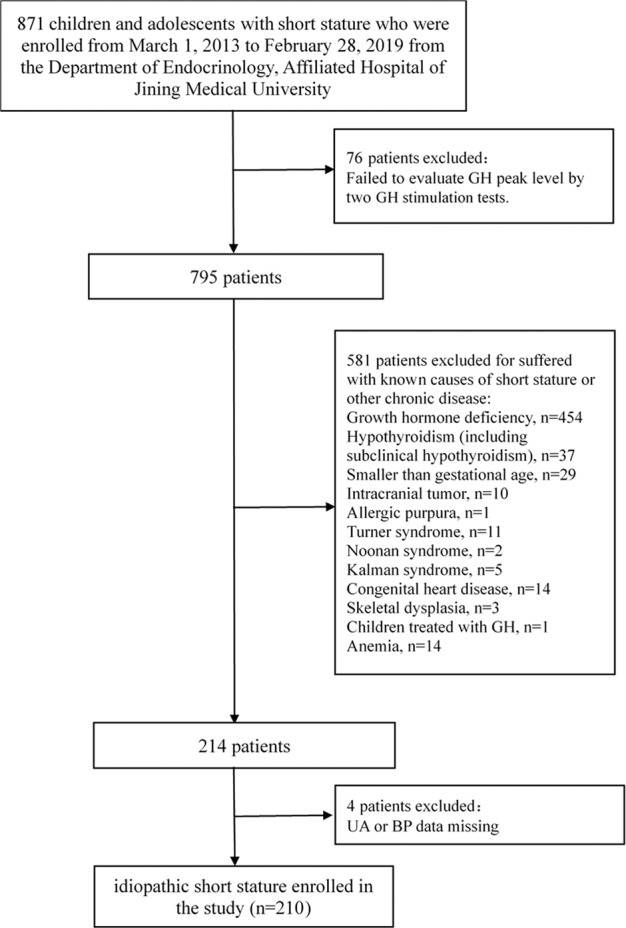
Flow chart of the study population. 871 children and adolescents with short stature were available during the study period and 210 children and adolescents with ISS were finally enrolled in the study.

This cross-sectional study was performed by reviewing the medical records of children and adolescents with short stature from the Department of Endocrinology, Affiliated Hospital of Jining Medical University between March 1, 2013 and February 28, 2019. ISS is a condition in which individuals in a similar living environment and of the same race, same sex and age who are two standard deviations lower than the average height of the normal population, without findings of identified causes of short stature by a complete evaluation by a paediatric endocrinologist including stimulated GH levels. Thus, according to previous literature [14, 15], the ISS criteria is described as follows: individuals which was more than two standard deviation scores (SDS) below the average of the same ethnic, age and sex; individuals with low growth velocity which means the growth velocity of children aged from 4.5 years to without the onset of puberty (in prepuberty) fail to reach 5 cm per year, or <6 cm per year for children with the onset of puberty (in puberty); individuals with normal birth length and weight; individuals with normal body proportions and body intake; individuals with a peak GH obtained in standard stimulation tests of at least 10 ng/ml; individuals without identified causes of short stature, such as dysmorphic syndromes, skeletal dysplasias, and systemic and endocrine diseases. The subjects were selected based on the following inclusion criteria: diagnosis of short stature, normal birth length and weight and a peak GH obtained in two GH stimulation tests of at least 10 ng/ml. The exclusion criteria included the following: participants with chronic organic diseases; participants diagnosed with growth hormone deficiency or other known causes of short stature; participants with chromosomal abnormalities, skeletal dysplasia, inherited metabolic diseases, thyroid dysfunction or abnormal liver function; and patients using drugs that interfere with GH secretion or function [14]. Thus, 871 patients were available during the study period, anthropomorphic and laboratory measurements of all participants were examined undertaken over 4 days to determine their diagnosis and causes of short stature. Finally, 210 children and adolescents with ISS (159 males and 51 females) aged 10.3 (3.9) years were eligible for our study and were **enrolled**, as described in the flow chart (Fig. 1).

### Anthropomorphic measurements

Heights were measured by a specially designated individual using the same measuring instrument (manufactured by Nantong Best Industrial Co., Ltd., Jiangsu, China). The height of each participant was measured in the morning with the participants without shoes, and the allowable error range was 0.1 cm. Height SDS was calculated based on the normal range of Chinese children [16]. The weight was measured with the participants in a fasted state using the same electronic scale (manufactured by Guangdong Xiangshan Weighing Apparatus Co., Ltd.) with an accuracy of ±0.1 kg. BMI was calculated as the ratio between the obtained body weight (kg) and height (m^2^). The detailed procedure for systolic and diastolic blood pressure (DBP) measurements is described as follows: The BP of the participants was measured using a standard sphygmomanometer after 5 min of seated rest on days 1 in the morning. The trained nurse then measured BP twice while the participant was seated and then calculated and recorded the mean of the two measurements of SBP and DBP, if the difference between two measurements was >5 mmHg, another measurement was performed and and then calculated and recorded the mean of the three measurements of SBP and DBP. According to Tanner stage [17], the stage of puberty was judged by physical examination. Prepubertal criteria are defined as follows: boys have a testicular volume of <4 mL and no pubic hair and girls have no breast development and no pubic hair [18, 19].

### Laboratory measurements

Blood sample was collected on days 2 in the morning after overnight fasting (at least 8 h). To assess GH secretion peaks, L-dopa (Levodopa Tablets, He Feng, Guang Xi, China; body weight more than 30 kg, 500 mg of levodopa; <30 kg, 250 mg of levodopa) was administered orally or insulin (Insulin Injection, Wan Bang, Jiang Su, China, 0.1 U/kg) was administered subcutaneously after overnight fasting. Blood samples were collected after 0, 30, 60, 90 and 120 min to obtain serum GH concentrations at each time point. GH was measured by the chemiluminescence method (ACCESS2, Beckman Coulter; USA) with an analytical sensitivity of 0.010 μg/L. Serum IGF-1 levels were measured by the chemiluminescence immunometric method (DPC IMMULITE 1000 analyser, SIEMENS, Germany) with intra- and inter-assay CVs for IGF-1 of 3.0% and 6.2%, respectively. Liver function index measurements including alanine aminotransferase and aspartate aminotransferase, renal function indicators including creatinine and uric acid (UA), and lipid profiles including total cholesterol (TC), triglyceride (TG), high-density lipoprotein cholesterol (HDL-C) and low-density lipoprotein cholesterol (LDL-C) and fasting blood glucose (FBG) were tested by a biochemical automatic analyser (Cobas c 702, Roche; Shanghai, China). Thyroid function, including free T3, free T4, thyroid-stimulating hormone, gonadotropin, cortisol rhythm and adrenocortical hormone, and urinary free cortisol (24UFC) were assessed using a luminescence immunoassay system (Cobas e 602, Roche; Shanghai, China).

### Statistical analysis

All analyses were performed with the statistical software packages R (http://www.R-project.org, The R Foundation) and EmpowerStats (http://www.empowerstats.com, X&Y Solutions, Inc, Boston, MA). We express continuous variables with a normal distribution as the mean (standard deviation) and continuous variables with a non-normal distribution as the median (quartile). Categorical variables are expressed in frequency or as a percentage. A univariate analysis model was used to determine the significance of the association between SBP and UA as well as the other independent variables. We then investigated the relationship between SBP and UA using smooth curve fitting after adjusting for potential confounders. Finally, we further applied a multivariate piecewise linear regression model to examine the threshold association between SBP and UA. *P* values < 0.05 (two-sided) were considered statistically significant.

## Results

### Baseline characteristics of selected participants

The clinical characteristics of all participants are described in Table 1. A total of 210 children and adolescents with ISS aged 10.3 (3.9) years were included in the study. According to previous literature [20], 22.85% of the subjects had UA elevation in the present study (UA > 5.5 mg/dl). The mean height SDS of the participants was −2.71 (0.55). Of the subjects, 159 (75.71%) were male. The majority of the children, 139 (66.19%), were prepubescent. The mean UA and SBP levels were 4.64 (1.35) mg/dl and 106.3 (12.4) mmHg, respectively.

**Table 1:** Baseline characteristics of participants.

Characteristics	All
No of participants	210
Age (years)	10.3 (3.9)
Male/Female (*N*)	159/51
Prepuberty/Puberty (*N*)	139/71
UA (mg/dl)	4.64 (1.35)
SBP (mmHg)	106.3 (12.4)
DBP (mmHg)	63.1 (9.7)
Height (cm)	126.3 (20.3)
Height SDS	−2.71 (0.55)
Weight (kg)	27.5 (11.6)
BMI (kg/m^2^)	16.45 (2.76)
BMI SDS	−0.53 (1.14)
IGF-1 (ng/ml)	215.74 (150.56)
IGF1 SDS	−0.66 (1.34)
TG (mmol/L)	0.68 (0.25)
TC (mmol/L)	3.75 (0.74)
HDL-C (mmol/L)	1.33 (0.26)
LDL-C (mmol/L)	2.03 (0.60)
ALT (U/L)	15.08 (6.42)
AST (U/L)	25.62 (6.62)
Cr (μmol/L)	39.85 (9.96)
BUN (μmol/L)	4.64 (2.14)
FBG (mmol/L)	4.78 (0.60)
24UFC (µg/dl)	135.52 (85.35)
Increased UA (*N*, %)	48 (22.85%)

*UA* uric acid, *SBP* systolic blood pressure, *DBP* diastolic blood pressure, *BMI* body mass index, *BMI SDS* the standard deviation score of BMI, *IGF-1* insulin-like growth factor-1, *IGF-1SDS* the standard deviation score of IGF-1, *TG* triglyceride, *TC* total cholesterol, *HDL-C* high-density lipoprotein cholesterol, *LDL-C* low-density lipoprotein cholesterol, *ALT* alanine aminotransferase, *AST* aspartate transaminase, *Cr* creatinine, *BUN* blood urea nitrogen, *FBG* fasting blood glucose, *24UFC* 24-h urinary free cortisol

### Association between SBP and anthropometrical and biochemical variables

Univariate linear regression analysis was performed to determine the relationships between clinical parameters and SBP. As shown in Table 2, for the unadjusted model, we observed a significant positive association between UA and SBP (*P* < 0.001). Other variables that remained significantly positively associated with SBP were age, BMI, IGF-1, 24UFC and pubertal stage (*P* < 0.05), whereas a significant negative association was found between SBP and sex (*P* < 0.05). No significant association was observed between SBP and TG, TC, HDL-C, LDL-C, or FBG (*P* > 0.05).

**Table 2:** Association between SBP (mmHg) and anthropometrical and biochemical variables.

Covariate	*β* (95%CI)	*P* value
Sex		
Male	Reference	
Female	−4.22 (−8.11, −0.33)	0.034
Age (years)	1.54 (1.16, 1.91)	<0.001
BMI SDS	1.10 (−0.37, 2.57)	0.144
BMI (kg/m^2^)	1.88 (1.33, 2.44)	<0.001
IGF-1 (ng/ml)	0.04 (0.03, 0.05)	<0.001
TG (mmol/L)	4.82 (−2.00, 11.64)	0.167
TC (mmol/L)	−1.45 (−3.77, 0.87)	0.222
HDL-C (mmol/L)	−1.49 (−8.07, 5.08)	0.656
LDL-C (mmol/L)	−1.42 (−4.28, 1.43)	0.329
FBG (mmol/L)	2.72 (−0.11, 5.55)	0.060
24UFC (µg/dl)	0.05 (0.03, 0.07)	<0.001
UA (mg/dl)	2.65 (1.46, 3.85)	<0.001
Tanner stage		
In prepuberty	Reference	
In puberty	10.42 (7.14, 13.69)	<0.001

*CI* confidence interval, *BMI SDS* the standard deviation score of BMI, *BMI* body mass index, *IGF-1* insulin-like growth factor-1, *TG* triglyceride, *TC* total cholesterol, *HDL-C* high-density lipoprotein cholesterol, *LDL-C* low-density lipoprotein cholesterol, *FBG* fasting blood glucose, *24UFC* 24-h urinary free cortisol, *UA* uric acid

### The independent association between SBP by multivariate piecewise linear regression

**Fig. 2:**
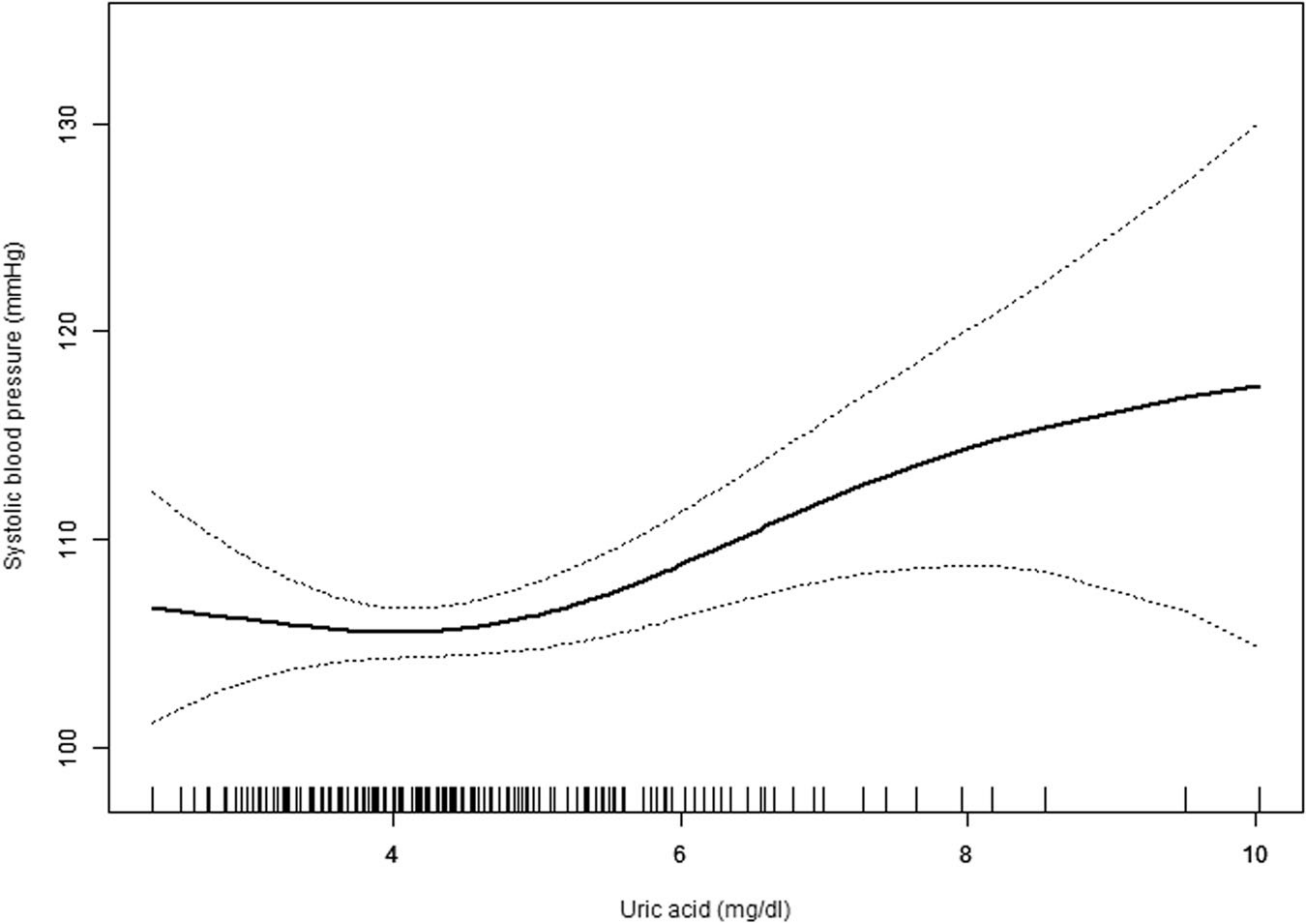
The relationship between UA and SBP by smooth curve fitting. Adjustment variables**:** age; sex; BMI body mass index; IGF-1 insulin-like growth factor-1, TG triglyceride, TC total cholesterol, HDL-C high-density lipoprotein cholesterol, LDL-C low-density lipoprotein cholesterol, FBG fasting blood glucose, 24UFC 24-h urinary free cortisol, Tanner stage.

As shown in Fig. 2, smooth curve fitting was performed after adjusting for possible confounding factors, including age, sex, BMI, IGF-1, TG, TC, HDL-C, LDL-C, FBG, 24UFC and Tanner stage. The participants’ SBP levels exhibited nonlinear relationships with UA, and the resulting curve exhibited a two-stage change and a breakpoint. When the UA level was greater than the breakpoint, there was a positive relationship between UA and SBP; however, if the value was less than the breakpoint, there was a negative relationship between UA and SBP. As shown in Table 3, we further analysed the threshold effect based on curve fitting, and the data indicated that the inflection point of UA was 4.13 mg/dl (95% CI 3.28, 6.65; *P* = 0.03). Specifically, SBP levels increased as UA increased when the UA level was >4.13 mg/dl (*β* 2.63, 95% CI 0.94, 4.31; *P* = 0.002). However, the SBP levels displayed a decreasing trend as UA increased when the UA level was <4.13 mg/dl, but the difference was not statistically significant (*β* −2.72, 95% CI −6.89, 1.45; *P* = 0.202).

**Table 3:** The independent association between SBP (mmHg) and UA (mg/dl) by multivariate piecewise linear regression.

Inflection point of UA	Effect size (*β*)	95% CI	*P* value
<4.13	−2.72	(−6.89, 1.45)	0.202
≥4.13	2.63	(0.94, 4.31)	0.002

Effect: systolic blood pressure.

Cause: uric acid.

Adjusted: age, sex, BMI body mass index, IGF-1 insulin-like growth factor-1, TG triglyceride, TC total cholesterol, HDL-C high-density lipoprotein cholesterol, LDL-C low-density lipoprotein cholesterol, FBG fasting blood glucose, 24UFC 24-h urinary free cortisol, Tanner stage

## Discussion

In this cross-sectional study, we observed a nonlinear relationship between UA and SBP in children and adolescents with ISS, and the UA turning point was 4.13 mg/dl (95% CI 3.28, 6.65; *P* = 0.03). The positive relationship between UA and SBP was significant only when the UA levels reached the inflection point.

Hyperuricaemia has been reported to have increased in children and adolescents in recent years. According to the known standards reported in the literature, a UA level > 5.5 mg/dl is considered abnormal [20]. According to this standard, in our study, the proportion of children and adolescents with hyperuricaemia reached 22.85%, suggesting that hyperuricaemia is common in ISS patients. The specific cause of elevated uric acid levels in children and adolescents in ISS is unclear, and we speculate that some of the underlying causes can be interpreted as follows: the final product of purine metabolism is uric acid, and dietary intake affects the production and metabolism of uric acid to a large extent. In our research process, we found that there are many unbalanced eating habits in ISS children and adolescents, such as picky eating, partial eating and excessive intake of high-sugar drinks, which may lead to an increase in uric acid levels [10].

Most previous literature has reported a linear positive association between UA and SBP in healthy adults and adolescents: The Framingham study found a significant positive association between UA levels and changes in SBP and DBP after 4 years in adults [21]. In children and adolescents, observational experiments have also drawn similar conclusions: Grayson et al. used a meta-analysis to show that hyperuricaemia is an independent risk factor for hypertension in healthy adolescents [22]. A study conducted in 6036 subjects aged 12–17 years in the United States showed that increased serum uric acid levels were associated with elevated BP in healthy adolescents and further revealed that a serum uric acid level > 5.5 mg/dl is associated with a twofold greater risk for hypertension [23]. Unlike previous literature reports, after adjusting for confounding factors related to SBP reported in the literature, such as BMI, sex, age and Tanner stage [24–26], we revealed a nonlinear relationship between UA and SBP in children and adolescents with ISS and the UA turning point was 4.13 mg/dl. In the previous literature, Feig et al. reported that UA level > 5.5 mg/dl is abnormal [20] and Sja’bani reported that UA level from 5 to 7 mg/dl is high-normal [27]. Unlike the previous definition of hyperuricaemia, we found that the SBP level was positively associated with UA when the UA values reached 4.13 mg/dl. As for the reason why we reached different conclusion from Feig’s and Sja’bani’s literature, we speculate that the heterogeneous of ethnic and different adjusted confounders may caused the difference. Our study was conducted in Chinese children and adolescents with ISS, so we should generalise our conclusions carefully to other populations with different characteristics. Further study are required to confirm its potential mechanism of the nonlinear relationship between UA and SBP levels in children and adolescents with ISS and provide clinical evidence for decreasing uric acid levels.

The present study has several limitations. First, our research was conducted in a homogeneous population of children and adolescents with ISS and the results cannot be extrapolated to other populations. Second, we did not collect data regarding other potential confounders of SBP, such as diet and family history. We intend to conduct a large-sample prospective study to specifically address factors affecting BP changes in children with ISS, including questionnaires about the dietary habits of the participants and the effects of a family history of hypertension. Third, the cross-sectional design of this study does not allow us to determine causality.

## Conclusion

In conclusion, we described a nonlinear relationship between UA and SBP levels in children and adolescents with ISS after adjusting for potential confounders and SBP levels were associated positively with the rise of UA levels when the UA levels reached the inflection point, however, it is uncertain whether the association is causal, and some relative future research are required to further confirm its potential mechanism and provide clinical evidence for controlling uric acid levels in children and adolescents with ISS.

### Summary table

#### What is known about the topic

- Short stature is considered a risk factor for adult hypertension, adult primary hypertension is likely derived from risk factors already present in childhood.
- Hyperuricaemia is a recognised risk factor for hypertension in adults and children.

#### What does this study add

- The univariate analysis results showed a significant positive association between uric acid and systolic blood pressure levels in children and adolescents with idiopathic short stature.
- There was a nonlinear relationship between uric acid and systolic blood pressure, and the uric acid turning point was 4.13 mg/dl, a multivariate piecewise linear regression model displayed a significant positive association between uric acid and systolic blood pressure when the uric acid levels more than 4.13 mg/dl.

## Data Availability

The datasets used and/or analysed in the current study are available from the corresponding authors upon reasonable request.
